# Brovalol, a New Valeric

**Published:** 1909-01

**Authors:** 


### BROVALOL, A NEW VALERIC.

Writing from Grawitz's Division of the Municipal Hospital Charlottenburg-Westend, Berlin, Dr. Maeder (*Therap. Monatshefte,* October, 1908,) states that for almost a year, the division has experimented on a large scale with a new valeric. It is a chemical compound of bromine, borneol and valerian, and contains 25.2 per cent. bromine, 48.3 per cent. borneol and 26.5 per cent. iso-valeric acid. The product is manufactured by the Schering Chemical Works and introduced in the United States, under the name of brovalol.

The remedy was used exclusively on patients with general nervous disturbances and proved to be a valuable sedative. The many complaints and difficulties of these patients,—mental and physical fatigue, insomnia, headache, etc.,—were in almost all cases decidedly benefited upon more or less long continued use. The remedy must be conceded to have a highly calmative, if not hypnotic action. In addition to its contents of bromine, brovalol has the material advantage of tasting much less disagreeably than the other valerics. It is distinguished by a mild taste and only a slight and aromatic odor. Because of this and of its tolerability, it was preferred by the patients to bornyval. Even large doses (single doses of 10 grams—2½ drams) were well borne, as animal experimentation showed.

